# Gambling Disorder in Parkinson’s Disease: A Scoping Review on the Challenge of Rehabilitation Strategies

**DOI:** 10.3390/jcm14030737

**Published:** 2025-01-23

**Authors:** Laura Culicetto, Federica Impellizzeri, Viviana Lo Buono, Giulia Marafioti, Giuseppe Di Lorenzo, Chiara Sorbera, Amelia Brigandì, Angelo Quartarone, Silvia Marino

**Affiliations:** IRCCS Centro Neurolesi “Bonino-Pulejo”, S.S. 113 Via Palermo C. da Casazza, 98124 Messina, Italy; laura.culicetto@irccsme.it (L.C.); viviana.lobuono@irccsme.it (V.L.B.); giulia.marafioti@irccsme.it (G.M.); giuseppe.dilorenzo@irccsme.it (G.D.L.); chiara.sorbera@irccsme.it (C.S.); amelia.brigandi@irccsme.it (A.B.); angelo.quartarone@irccsme.it (A.Q.); silvia.marino@irccsme.it (S.M.)

**Keywords:** gambling disorder, rehabilitation, Parkinson, DBS, CBT

## Abstract

**Background**: Pathological gambling (PG) is a significant non-motor complication in Parkinson’s disease (PD), often linked to dopaminergic therapies. PG impacts the quality of life of patients and their families, presenting unique challenges due to its interplay with motor symptoms and cognitive impairments in PD. This scoping review synthesized current evidence on rehabilitation strategies for PG in PD. **Methods**: A systematic review was conducted across the PubMed, Web of Science, Embase, and Scopus databases to identify studies addressing pharmacological, psychological, and neuromodulatory approaches. The scoping objective was to map the available evidence on treatment strategies and identify research gaps. **Results**: Of 537 studies, 5 met the inclusion criteria. Pharmacological strategies included dopamine agonist adjustments, naltrexone, and amantadine, which showed partial efficacy but were often limited by significant side effects. Neuromodulation via deep brain stimulation (DBS) demonstrated potential by modulating limbic circuits, though risks like apathy and transient symptom exacerbation were noted. Psychological interventions, particularly cognitive-behavioral therapy (CBT), were the most effective in modifying gambling behaviors, though adaptations are needed for PD-specific cognitive and psychiatric challenges. **Conclusions**: Effective rehabilitation for PG in PD requires a multidisciplinary approach to balance motor symptom management with impulse control disorders. While pharmacological, psychological, and neuromodulatory interventions show promise, gaps remain regarding their long-term efficacy, combined use, and tailoring to PD-specific needs. The lack of large-scale, comparative studies and limited exploration of novel therapeutic combinations underscore the need for further research to optimize patient outcomes and develop comprehensive treatment frameworks.

## 1. Introduction

Parkinson’s disease (PD) is a progressive neurodegenerative disorder primarily characterized by motor symptoms such as tremor, rigidity, and bradykinesia [1,2]. However, non-motor symptoms, including cognitive and behavioral disorders, have garnered increasing attention in recent years [3].

Pathological Gambling (PG), also known as gambling disorder, is defined as a persistent and recurrent maladaptive pattern of gambling behavior that leads to significant impairment or distress (American Psychiatric Association, 2013) [4,5,6,7]. Behavioral addictions like PG are driven by repetitive impulsive actions that severely disrupt the lives of both patients and their families. Key characteristics include cognitive salience (domination of thoughts and behaviors by the activity), conflicts with other life aspects, temporary euphoria or relief, tolerance, withdrawal symptoms, and relapse. In PD patients, these behaviors are exacerbated by cognitive deficits, particularly those affecting executive functions and decision-making, as well as by psychiatric symptoms, which further intensify gambling tendencies [7]. Notably, the prevalence of PG in patients with PD ranges from 2.2% to 7% in tertiary PD clinics, a figure notably higher than that observed in the general population [7,8,9,10]. Consequently, the burden of these symptoms negatively affects the quality of life for both patients and their caregivers [8].

The pathophysiology of PG in PD is closely associated with dopaminergic treatments, particularly dopamine agonists [9]. While these therapies effectively alleviate motor symptoms, they can overstimulate the mesolimbic pathway, a key component of the brain’s reward system, thereby increasing the risk of developing impulsive–compulsive disorders (ICDs), including gambling disorder [10,11]. This overstimulation often results in an “overdose” of ventral striatal-cortical circuitry, leading to impulsive–compulsive behaviors, such as PG [12].

These behaviors are driven by ongoing dopaminergic activation, which sensitizes the ventral striatal system and manifests as an increased drive for maladaptive behaviors. Moreover, impaired learning from negative outcomes and altered decision-making processes further exacerbate these tendencies. Dopaminergic therapies may impair the balance between reward and punishment processing in PD patients, reducing their ability to appropriately regulate behaviors and reinforcing compulsive gambling [13].

This dual-edged nature of dopaminergic therapy poses significant challenges in the clinical management of PD, particularly as impaired decision-making amplifies the risk of developing gambling behaviors [14].

Managing gambling disorder in PD is particularly complex due to the dual challenge of addressing both gambling behaviors and PD symptoms. While pharmacological treatments, behavioral therapies, and cognitive interventions have been proposed, there is limited evidence describing their application and adaptability in this specific context [15,16]. Further exploration of tailored strategies that address the unique needs of PD patients with gambling disorders is essential for improving patient outcomes and guiding future research.

This scoping review addresses the growing need for targeted rehabilitation and behavioral management strategies tailored to individuals with PD and PG.

Despite an expanding body of research, there is a lack of comprehensive synthesis on rehabilitation strategies for PG in PD. Given the increasing recognition of PG as a significant non-motor complication in PD, this scoping review aims to consolidate the current evidence on rehabilitation strategies, identify knowledge gaps, and provide practical guidance for future research. By synthesizing this information, the review seeks to provide clinicians with practical guidance for managing PG in PD, ultimately enhancing clinical decision-making and patient care.

## 2. Materials and Methods

A scoping review was conducted to investigate rehabilitation and treatment strategies for PG in PD. This review adhered to the PRISMA extension for scoping reviews (PRISMA-ScR) guidelines to ensure methodological rigor [17] and was registered on OSF (10.17605/OSF.IO/JC6RK) in December 2024, after the search was conducted in August 2024 and prior to manuscript submission.

### 2.1. PCC Model

The PCC framework (Population, Concept, and Context), as recommended by Pollock et al. [18], was employed to construct clear and meaningful objectives and eligibility criteria for this scoping review:

Population: Individuals diagnosed with PD exhibiting PG behaviors.

Concept: Rehabilitation strategies, including pharmacological treatments, neuromodulation techniques, cognitive-behavioral therapies, and other therapeutic approaches aimed at managing PG.

Context: Studies were conducted across various healthcare and rehabilitation settings, with no geographical or cultural restrictions.

### 2.2. Search Strategy

A scoping review of currently published studies was performed in August 2024 using the following databases: Scopus, PubMed, Web of Science, and Embase. The search combined the following terms: (“Parkinson’s Disease” OR “Parkinson Disease” OR “PD”) AND (“Gambling Disorder” OR “Pathological Gambling” OR “Compulsive Gambling”) AND (“Rehabilitation” OR “Therapy” OR “Treatment” OR “Intervention”) AND (“Pharmacological Treatment” OR “Medication” OR “Drug Therapy” OR “Pharmacotherapy” OR “Psychological Treatment” OR “Cognitive Behavioral Therapy” OR “CBT” OR “Neurostimulation” OR “Neuromodulation” OR “Deep Brain Stimulation” OR “DBS”). No restriction was placed on the publication year of the article.

#### Inclusion and Exclusion Criteria

The inclusion criteria were (i) adult patients with PG and PD; (ii) studies described rehabilitation interventions, including pharmacological or psychological treatments for gambling disorder; (iii) studies assessing gambling disorders using neuropsychological tests or clinical interviews; (iv) studies exploring the impact of gambling rehabilitation in PD; (v) peer-reviewed articles published in English, (vi) studies conducted in healthcare or rehabilitation settings, with no geographical or cultural restrictions.

We excluded (i) conference proceedings or commentary; (ii) review; (iii) letters to the editor, and (iv) grey literature such as conference proceedings, reports, and non-peer-reviewed literature. This exclusion was made to ensure methodological rigor by focusing on peer-reviewed articles, which are considered to offer a higher level of quality and reliability.

### 2.3. Study Selection

To minimize bias and ensure a rigorous selection process, two authors (L.C. and F.I.) independently screened the titles and abstracts of the studies based on the inclusion and exclusion criteria. Any discrepancies were resolved through collaborative discussion, with consultation from a third author (V.L.B). This multi-step approach ensured that at least three researchers independently assessed each article. The final inclusion or exclusion decision was based on predefined criteria regarding population (e.g., PD patients with PG behaviors), concept (e.g., rehabilitation interventions), and context (e.g., healthcare or rehabilitation settings). In cases of persistent disagreement, the final decision involved all authors.

### 2.4. Data Extraction and Analysis

The studies that met the inclusion criteria were summarized based on the following points: (1) study characteristics, including the type of study and the country where the data were collected; (2) patient characteristics, such as the sample size, age, gender, duration of disease, and the level of education; and (3) key findings and relevant outcomes.

Following the full-text selection, data were extracted from the included studies and reported in a table using Microsoft Excel (Version 2021). The extracted data included study title, first author name, year of publication, study aims and design, sample size, type of participants, type of intervention and control, baseline performance, type of outcome and time-points for assessment, results, and key conclusions.

Moreover, the agreement between the two reviewers (L.C. and F.I.) was assessed using the kappa statistic. The kappa score, with an accepted threshold for substantial agreement set at >0.61, was interpreted to reflect substantial concordance between the reviewers. This criterion ensures a robust evaluation of the inter-rater reliability, ensuring a high level of agreement in the data extraction process.

## 3. Results

**Figure 1:**
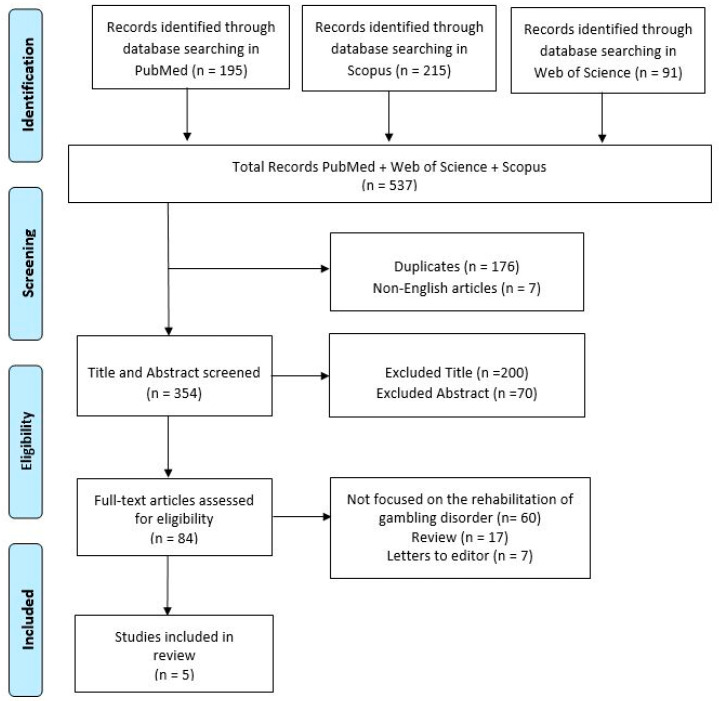
Search and selection of eligible articles.

The study selection process adhered to the PRISMA-ScR guidelines and is summarized in Figure 1. Of the 537 studies initially identified through database searches, 354 remained after the duplicate removal. Following the title and abstract screening, 84 studies were assessed for eligibility based on their full text. Ultimately, five studies were included in the review, meeting all predefined eligibility criteria. The detailed search strategies used in each database are provided as Supplementary Material.

### 3.1. Characteristics of Included Studies

The five studies represented a range of methodologies and populations. Key details include the following: Study designs: the studies employed various designs, including randomized controlled trials and observational studies, focusing on interventions for PG in PD. Participants: sample sizes ranged from 2 to 30 participants, with a majority being male, and the mean ages of participants varied between 43 and 61 years old, and disease durations ranged from 5 to 7 years. Interventions: interventions included pharmacological treatments (e.g., naltrexone and amantadine), neuromodulation (e.g., subthalamic nucleus deep brain stimulation [STN-DBS]), and cognitive-behavioral therapy (CBT), although most studies tailored their approaches to the specific challenges associated with PG in PD. Outcomes: gambling behaviors were assessed using validated tools such as the South Gambling Screen (SOGS) and diagnostic interviews based on DSM-IV criteria, additional assessments included cognitive tests (e.g., Mini-Mental State Examination, MMSE) and emotional evaluations (e.g., Beck Depression Inventory).

### 3.2. Key Findings

PG in PD is a recognized non-motor complication frequently associated with dopaminergic therapy. Treatment approaches, including behavioral and pharmacological interventions, have shown varying degrees of success in managing PG symptoms in PD patients. Several studies have explored the efficacy of these interventions, with particular focus on CBT, deep brain stimulation (DBS), and pharmacological treatments (Table 1).

Jiménez-Murcia [19] demonstrated that CBT effectively treats PG in both PD and non-PD populations, though PD patients exhibited slightly higher rates of relapse and dropout. Most pathological gamblers in this study were male, with primary issues related to slot machines and bingo, and an average problem duration of five years. Pathological gamblers with Parkinson’s disease (PG + PD) exhibited lower rates of smoking and employment compared to their non-PD counterparts (PG − PD). DBS of the subthalamic nucleus (STN) has also been investigated as a treatment for non-motor symptoms, including PG. Evidence suggests that STN-DBS may reduce impulsive behaviors associated with PG by desensitizing the limbic dopamine system, while simultaneously decreasing the need for dopaminergic therapy [20]. In one study, six patients experienced resolution of PG symptoms following STN-DBS; however, two patients experienced an exacerbation of PG due to postoperative mania. Additionally, three patients had transient episodes of depression that triggered gambling behavior, although the pleasure derived was reportedly less intense than before. Long-term follow-up revealed increased apathy scores, with two patients developing pathological apathy [21]. Pharmacological treatments have also shown promise. Amantadine, an anti-glutamatergic drug with N-methyl D-aspartate (NMDA) receptor antagonist properties, has been studied for its effects on PG in PD patients. A double-blind, placebo-controlled crossover study involving 17 patients with PD demonstrated that amantadine could reduce or abolish PG symptoms within 2 to 3 days. However, side effects such as hallucinations and confusion were reported [22]. Additionally, the opioid antagonist naltrexone has shown promise in treating PG. Additionally, the opioid antagonist naltrexone has emerged as a potential treatment for PG. In one study, naltrexone led to significant remission of PG symptoms in PD patients, though liver abnormalities were observed in some cases. These side effects resolved after dosage adjustment, suggesting that naltrexone may be an effective and well-tolerated option for managing PG in PD [23]. These studies highlight a variety of treatment strategies for PG in PD, demonstrating the potential of both behavioral and pharmacological approaches. However, side effects and individual variability must be carefully considered when designing treatment plans for this population.

## 4. Discussion

This scoping review examines rehabilitation approaches for PG in PD, a multifaceted behavioral complication often linked to dopaminergic therapy. PG in PD patients has profound implications, affecting both patients themselves and their families, which underscore the importance of developing management strategies that address both behavioral and motor symptoms.

Key risk factors for developing PG in PD include male gender, early onset of PD, and a personal or family history of gambling problems, as well as alcohol and/or substance abuse [24,25,26]. Additionally, higher caffeine and cigarette consumption, motor complications, and greater peak doses of dopamine agonists have been identified as significant predictors of ICDs [27]. Identifying these risk factors is crucial for recognizing high-risk patients and tailoring preventive and therapeutic strategies. Reflecting this evidence, most studies reviewed included a higher proportion of male patients, consistent with previous findings. This gender disparity may be influenced by biological, psychological, and sociocultural factors, such as differing patterns of dopamine receptor sensitivity and risk-taking behaviors between men and women. Men are also more likely to engage in behaviors such as gambling, which may partially explain their overrepresentation in studies focusing on PG in PD [28,29].

Dopaminergic therapy remains strongly associated with the onset of PG [30]. Gambling behaviors frequently emerge following the initiation of dopamine agonists or other dopaminergic agents. Jiménez-Murcia et al. [19] observed this phenomenon in PD patients, aligning with evidence that implicates dopaminergic overstimulation of the mesolimbic pathway, a critical reward-processing circuit. While these therapies effectively manage motor symptoms, they may inadvertently promote impulsive behaviors, including PG. Interestingly, PG in PD typically manifests later in life compared to non-PD gamblers, whose gambling behaviors often begin in youth. This delayed onset may reflect the combined effects of dopaminergic therapy and age-related cognitive vulnerabilities [31,32].

Pharmacological strategies for PG management often involve adjusting dopaminergic therapy or introducing alternative treatments. L-dopa, combined with monoamine oxidase B inhibitors, remains a first- and second-line treatment option for PG in PD, particularly in cases requiring dopamine agonist withdrawal [33].

PG symptoms frequently resolve partially or completely following discontinuation of dopamine agonists; however, some cases persist despite these adjustments. For instance, Bosco et al. [23] demonstrated the efficacy of naltrexone, an opioid antagonist that modulates the reward circuitry, particularly the mesolimbic pathway, in patients with persistent PG, emphasizing its role in managing reward-related behaviors. However, outcomes with opioid antagonists have been variable, highlighting the need for further research to confirm their efficacy [34]. Similarly, while Selective Serotonin Reuptake Inhibitors (SSRIs) or low-dose clozapine [35] have shown benefits in some cases, others report persistent symptoms or severe consequences, including suicide following relapse [36]. These findings highlight the importance of personalized approaches, particularly for patients who cannot or will not modify their dopaminergic therapy. Preliminary studies on medications such as zonisamide and topiramate have shown promising results in reducing gambling urges, but more robust clinical trials are needed to validate these findings [37,38].

Amantadine, another pharmacological option, has demonstrated rapid effects in reducing gambling behaviors, typically within 2–3 days [22]. Its mechanism likely involves restoring the glutamate/dopamine balance. However, its use is often limited by side effects such as hallucinations, confusion, and psychosis, leading to discontinuation in nearly 30% of cases [39,40]. Moreover, the Movement Disorders Society Evidence-Based Medicine (MDS-EBM) review found insufficient evidence supporting amantadine’s efficacy for PG in PD [41]. These limitations necessitate careful patient selection and monitoring.

Neuromodulation techniques such as DBS, have emerged as a potential alternative for addressing PG in PD. DBS targets motor and limbic areas of the subthalamic nucleus (STN), mitigating hyperdopaminergic states linked to impulsive behaviors [21]. DBS may also indirectly influence corticostriatal plasticity, reshaping reward-related circuits [42]. However, these benefits come with potential risks, including transient postoperative exacerbation of PG and long-term apathy due to reduced dopaminergic tone. Close, long-term follow-up is essential to monitor motivational and behavioral states in DBS-treated patients.

Psychological interventions encompass a range of options, including full-length, professionally delivered therapies such as behavior therapy, cognitive therapy, and CBT, as well as self-directed approaches like workbooks and computer-facilitated programs. These flexible alternatives cater to individuals seeking tailored treatment solutions. Motivational approaches, including motivational interviewing and motivational enhancement therapy, have also shown significant effectiveness, particularly for individuals experiencing less severe gambling issues [43].

However, CBT remains the gold standard for PG treatment, with evidence supporting its efficacy in modifying maladaptive beliefs and behaviors [44,45]. CBT incorporates components such as cognitive restructuring, problem-solving skills, and social skills training, targeting superstitious beliefs, overestimation of gambling skills, and impulsivity. Long-term benefits lasting 6–12 months post-therapy have been reported, positioning CBT as a cornerstone of PG management [45]. However, CBT in PD patients presents unique challenges, including cognitive deficits (e.g., memory, attention, and language impairments) and psychiatric comorbidities. Tailoring CBT to address these needs may reduce relapse and dropout rates while enhancing treatment outcomes. Further, although the studies included in our review did not specifically examine cognitive group therapy, existing literature indicates its effectiveness in reducing pathological gambling [46].

Family involvement is critical for maximizing the effectiveness of interventions. Family members should be encouraged to limit the patient’s access to money, credit cards, and the Internet while actively participating in therapeutic programs. These measures can help families better understand the disorder, develop effective coping strategies, and provide supportive environments that complement the patient’s treatment plan [47].

Emerging therapies such as digital health solutions, including mobile apps and wearable devices for tracking and managing impulsive behaviors, and virtual reality-based cognitive training, are showing potential as complementary interventions for PG in PD [48,49,50,51]. These innovative approaches leverage technology to improve therapeutic engagement, enhance adherence to treatment protocols, and address cognitive and behavioral deficits in ways that can be tailored to patient-specific needs. For instance, mobile apps can provide real-time monitoring and feedback, promoting self-management and reducing impulsivity, while virtual reality (VR) training offers immersive environments for cognitive rehabilitation, offering patients interactive and engaging tasks that can be adjusted to their individual capabilities [48]. While evidence is still emerging, incorporating these modalities into future research may help address current gaps in treatment strategies and optimize outcomes.

The studies reviewed used various methods to assess gambling disorder, ranging from self-administered questionnaires to clinician-led diagnostic interviews. This variability may contribute to inconsistencies in identifying and reporting gambling behaviors. For instance, self-administered tools like the South Oaks Gambling Screen (SOGS) often underestimate prevalence compared to clinical interviews based on DSM-IV criteria [16]. This discrepancy underscores the need for validated diagnostic tools that involve both patients and caregivers, such as the Questionnaire for Impulsive–Compulsive Disorders in Parkinson’s Disease (QUIP) and the Dopamine Dysregulation Syndrome–Patient and Caregiver (DDS-PC) inventory. Additionally, traits such as aggressiveness, anxiety, and disinhibition are more commonly associated with PG in PD than in non-gamblers [52]. Future studies should prioritize comprehensive psychological assessments to better understand these associations.

This scoping review reveals substantial heterogeneity in PG presentations and treatment responses in PD. While pharmacological treatments, DBS, and CBT demonstrate varying degrees of efficacy, each approach has limitations and risks. Furthermore, the lack of large-scale, comparative studies evaluating these interventions highlights a critical gap in the literature. Comparative studies are crucial for identifying the relative effectiveness of these approaches and for guiding personalized, evidence-based treatment strategies tailored to patient needs. Future research should prioritize rigorous comparative trials across diverse patient populations to address this gap and identify optimal strategies for managing PG in PD. These efforts will ultimately guide clinical decision-making and improve patient outcomes.

In conclusion, the findings of this review provide valuable insights for clinical practice by highlighting rehabilitation strategies for PG in PD.

To integrate interventions into standard care for PD patients, a multidisciplinary approach involving neurologists and psychologists is essential. Pharmacological treatments like dopaminergic therapy adjustments or medications such as naltrexone can be personalized based on patient needs. CBT should be adapted to address cognitive deficits and comorbid conditions and integrated into care with mental health professionals.

DBS, though mainly for motor symptoms, can be considered for severe, treatment-resistant PG with careful monitoring of both motor and non-motor effects.

Emerging technologies like mobile apps and VR can complement PG management by aiding real-time behavior tracking and providing cognitive rehabilitation. These innovations should be integrated into care with provider involvement to enhance existing treatments. Incorporating these personalized, multidisciplinary strategies into routine clinical practice can improve the management of PG in PD, leading to better outcomes and quality of life for patients.

By addressing the interplay between motor symptoms and impulsive behaviors, this review underscores the need for personalized and multidisciplinary interventions. These strategies, when incorporated into routine clinical practice, can optimize the management of PG in PD, ultimately improving patient outcomes and quality of life.

## 5. Limitations

The scoping review process itself has inherent limitations that may influence the findings. Firstly, although we followed a rigorous methodology, the subjectivity involved in interpreting and applying inclusion/exclusion criteria could introduce bias. Secondly, even though we conducted comprehensive research, relevant studies may have been missed due to limitations in search terms or database coverage. Specifically, the exclusion of grey literature may have limited the comprehensiveness of the review, as valuable information from non-peer-reviewed sources was not included. Furthermore, the search terms used, particularly for interventions, may have been too narrow (e.g., using “deep brain stimulation” instead of broader terms like “stimulation”), potentially excluding studies with relevant interventions that did not specifically match these terms. Additionally, the heterogeneity of the included studies, particularly in terms of design, population, and intervention type, limited direct comparisons and hindered the synthesis of consistent conclusions. Furthermore, despite employing a multi-reviewer approach to minimize bias, the data extraction and synthesis processes may have been influenced by reviewer interpretations. Lastly, as per the scoping review methodology, we did not conduct a critical appraisal of study quality, which may impact the reliability of the synthesized evidence. The selected studies have several limitations, most notably their small sample sizes, which reduce the generalizability of the findings. Many of these studies included only 3 to 30 participants, inherently lacking statistical power to draw robust conclusions applicable to broader populations. Additionally, some studies exhibited limited diversity in their participant pools, such as including only male participants or highly specific subgroups, further restricting the applicability of the results. These limitations highlight the need for future research to prioritize larger and more diverse cohorts, which would improve the reliability and generalizability of the evidence for clinical application. Another significant limitation is the lack of long-term follow-up in the included studies. Most studies did not assess the sustained impact of the interventions over time, limiting our understanding of their long-term effectiveness. Given the nature of PG and the chronic course of PD, future studies should incorporate long-term follow-up to evaluate the durability of the treatment effects. This would be important for understanding whether the benefits of interventions are sustained and whether new therapeutic strategies may be necessary over time. Many of the included studies relied on self-reported data (e.g., [19]), which are inherently prone to biases such as recall bias. This occurs when participants inaccurately recall past behaviors or experiences, potentially leading to overestimation or underestimation of their gambling habits or related symptoms. The subjective nature of self-reported data is particularly problematic in studies addressing sensitive issues like PG, where factors such as social desirability or stigma may further distort responses. These limitations compromise the reliability of the findings, particularly in the context of comorbid psychiatric disorders like depression, anxiety, or substance use disorders, which were often underreported or inconsistently assessed. To enhance the validity of future research, it is essential to incorporate objective measures, such as clinical assessments or behavioral tracking, and to conduct comprehensive evaluations of comorbidities using standardized diagnostic tools.

## 6. Conclusions

While this review highlights potential rehabilitation strategies for managing PG in PD, the limited number of studies included reduces the certainty of these findings. Effective management of PG in PD requires a comprehensive and multifaceted approach that combines motor symptom control with behavioral rehabilitation. Pharmacological treatments such as amantadine and naltrexone, neuromodulation techniques like DBS, and psychological interventions such as CBT each offer unique benefits. However, their implementation must account for the complexity of PD, including cognitive impairments, psychiatric comorbidities, and the heterogeneity of PG manifestations. Despite the promising nature of these therapies, their implementation in real-world clinical settings presents several challenges. Patient adherence to treatment plans can be hindered by the cognitive and emotional complexities often present in individuals with PD and PG. Additionally, financial constraints may limit access to specialized rehabilitation programs and neuromodulatory techniques, particularly in resource-limited settings. Furthermore, successful implementation often requires a multidisciplinary approach involving neurologists, psychiatrists, psychologists, and rehabilitation specialists, which can be difficult to coordinate. Addressing these challenges will require targeted strategies, including patient education, funding support, and enhanced collaboration across disciplines. To optimize patient outcomes, it is essential to conduct high-quality, large-scale studies to better understand the efficacy and safety of these interventions. Future research should also prioritize developing tailored interventions and exploring the long-term effectiveness of combined treatment strategies. Addressing these gaps will not only enhance clinical decision-making but also improve the quality of life for PD patients and their families.

## Figures and Tables

**Table 1:** Main characteristics of the included studies.

Study	Population	Duration of PG	Test Gambling	Cognitive or Motor Test	Emotional Test	Intervention	Results
Jiménez-Murcia 2012	30 patients (47.9 ± 11.5)	Mean duration 5 years	SOGS; Diagnostic Questionnaire for PG based on DSM-IV.	NA	SCL-90-R	CBT. The intervention lasted 16 weeks, with weekly 45-min sessions conducted by experienced psychologists and psychiatrists	CBT was effective for PG in both PD and non-PD patients, highlighting the importance of tailoring treatment to the specific needs of PD patients
Bosco 2012	3 male patients (from age 43 to 51)	NA	Clinical interview according to the DSM-IV	UPDRS	NA	Naltrexone. Daily administration of naltrexone at doses starting from 50 mg per day for 6 months, managed by neurologists and psychiatrists	naltrexone effectively reduced or resolved persistent PG in PD patients unresponsive to dopamine agonist adjustments, with minimal side effects
Ardouin 2006	7 (6 males) (54 ± 9)	Mean of 7 years, associated with dopaminergic treatment	Clinical interview according to the DSM-IV	Mattis Dementia Rating Scale; UPDRS	BDI; Starkstein Apathy Scale	STN DBS. The intervention involves STN-DBS over an average follow-up of 3.4 years, administered by neurosurgeons and neurologists	STN-DBS resolved PG in all patients by reducing dopaminergic medication, with improvements observed within 18 months
Thomas 2010	17 (13 males) 61.0 ± 1.6	7.1 ± 0.4	SOGS; G-SAS; Y-BOCS	MMSE, Neuropsychiatry Inventory	NA	Amantadine. The intervention involved daily administration of amantadine at 100 mg twice a day for 3 months, managed by neurologists	Amantadine reduced or eliminated PG symptoms though side effects like hallucinations and confusion led to discontinuation in nearly 30% of cases.
Bandini 2007	2 males; 51 and 43 years	NA	Clinical interview according to the DSM-IV	MMSE	HAM-D	STN-DBS. The intervention involved continuous bilateral STN-DBS with dopaminergic medication reduction, managed by neurosurgeons and neurologists, with follow-up extending up to 6 months	STN-DBS, combined with a significant reduction in dopaminergic therapy, effectively resolved PG in PD patients within 1–2 months

Legend: SOGS: South Oaks Gambling Screen; CBT: Cognitive Behavioral Therapy; SCL-90-R: Symptoms Checklist-90 items-Revised; STN-DBS: subthalamic nucleus deep brain stimulation; BDI: Beck Depression Inventory; DSM: Diagnostic and Statistical Manual of Mental Disorders; UPDRS: Unified Parkinson’s Disease Rating Scale; G-SAS: Gambling Symptom Assessment Scale; Y-BOCS: Yale–Brown Obsessive-Compulsive Scale; MMSE: Mini-Mental State Examination; HAM-D: Hamilton Depression Rating Scale; NA: Not available.

## Supplementary Materials

The following supporting information can be downloaded at: www.mdpi.com/article/10.3390/jcm14030737/s1. File S1: search strategy utilized for each database.
