# Supplementary material for: Gambling Disorder in Parkinson’s Disease: A Scoping Review on the Challenge of Rehabilitation Strategies
Source: J Clin Med. 2025 Jan 23;14(3):737. doi: 10.3390/jcm14030737 (PMC11818531; doi:10.3390/jcm14030737)
Supplement: Supplementary file 1 [file jcm-14-00737-s001.zip › jcm-3409591-File S1.pdf]

Below is the search strategy utilized for each database:

Scopus: TITLE-ABS-KEY ( ( "Parkinson's Disease" OR "Parkinson Disease" OR "PD" ) AND ( "Gambling Disorder" OR "Pathological Gambling" OR "Compulsive Gambling" ) AND ( "Rehabilitation" OR "Therapy" OR "Treatment" OR "Intervention" ) AND ( "Pharmacological Treatment" OR "Medication" OR "Drug Therapy" OR "Pharmacotherapy" OR "Psychological Treatment" OR "Cognitive Behavioral
Therapy" OR "CBT" OR "Neurostimulation" OR "Neuromodulation" OR "Deep Brain Stimulation" OR "DBS" ) )

PUBMED: ("Parkinson's Disease" OR "Parkinson Disease" OR "PD") AND ("Gambling Disorder" OR "Pathological Gambling" OR "Compulsive Gambling") AND ("Rehabilitation" OR "Therapy" OR "Treatment" OR "Intervention") AND ("Pharmacological Treatment" OR "Medication" OR "Drug Therapy" OR "Pharmacotherapy" OR "Psychological Treatment" OR "Cognitive Behavioral Therapy" OR "CBT" OR "Neurostimulation" OR "Neuromodulation" OR "Deep Brain Stimulation" OR "DBS")

WEB OF SCIENCE: ALL=(("Parkinson's Disease" OR "Parkinson Disease" OR "PD") AND ("Gambling Disorder" OR "Pathological Gambling" OR "Compulsive Gambling") AND ("Rehabilitation" OR "Therapy" OR "Treatment" OR "Intervention") AND ("Pharmacological Treatment" OR "Medication" OR "Drug Therapy" OR "Pharmacotherapy" OR "Psychological Treatment" OR "Cognitive Behavioral Therapy" OR "CBT" OR "Neurostimulation" OR "Neuromodulation" OR "Deep Brain Stimulation" OR "DBS"))
